# Enantioselective Synthesis of Homo-*N*-Nucleosides Containing a 1,4-Dioxane Sugar Analog

**DOI:** 10.3390/molecules13122962

**Published:** 2008-12-03

**Authors:** Qiang Yu, Per Carlsen

**Affiliations:** Department of Chemistry, Norwegian University of Science and Technology (NTNU), N-7491 Trondheim, Norway; E-mail: qiang@nt.ntnu.no

**Keywords:** Sugar analog, Nucleoside analogs, Homo-*N*-Nucleoside, 1,4-Dioxane, Hetero-cycles.

## Abstract

A dioxane homo-sugar analog, (2*S*,5*S*)-and (2*R*,5*S*)-5-[(4*S*)-2,2-dimethyl-1,3-dioxolan-4-yl]-2-iodomethyl-1,4-dioxane was prepared from (2*R*,3*R*)-dimethyl tartrate, and further elaborated into the corresponding homo-*N*-nucleoside analogs by its reactions with uracil and adenine, respectively.

## Introduction

**Figure 1:**
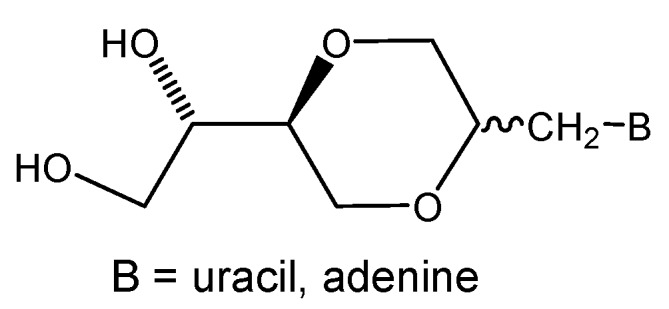
A representative structure of the 1,4-dioxane homo-*N*-nucleoside analogs.

There has been an increasing interest in the synthesis of nucleoside analogs with modifications of the sugar moiety for the purpose of obtaining new antiviral and antitumor agents [1,2,3]. A well known class of modified nucleosides are the homo-*N-* and *C*-glycosidic nucleosides [4,5,6,7,8] The insertion of a methylene group between the heterocyclic base and the sugar moiety results in a more flexible structure, and due to the lack of an anomeric acetal position, these nucleosides are in general resistant to enzymatic degradation [8]. For these reasons, we decided to pursue the synthesis of nucleoside analogs, however, based on the conformationally more flexible, optically active homo-1,4-dioxane sugar analogs. The objective was thus to construct novel homo-*N*-nucleoside analogs containing a 1,4-dioxane sugar moiety. A representative structure is shown in Figure 1, where the 1,4-dioxane homo-sugar analog is substituted with uracil or adenine, respectively.

## Results and Discussion

**Scheme 1:**
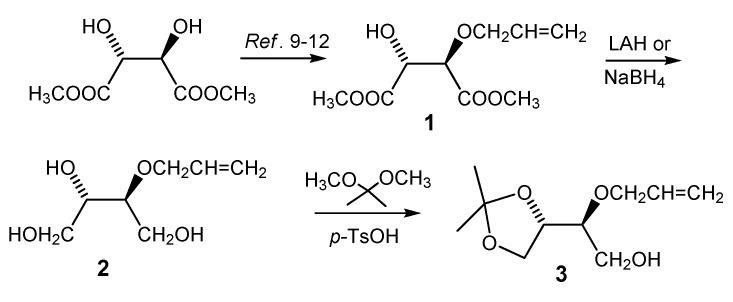
Synthesis of the partially protected (2*S*,3*S*)-2(allyloxy)butane-1,3,4-triol, **3**.

The formation of the 1,4-dioxane homo-sugar analog **4** was readily achieved starting from (2*R*,3*R*)-dimethyl tartrate, an inexpensive and commercial available chiral starting material. Thus, (2*R*,3*R*)-dimethyl tartrate was converted into the corresponding enantiomerically pure allyl ether **1** [9] either by the reaction with allyl bromide in the presence of silver oxide [10] or in a tin assisted reaction with dibutyltin oxide [11,12]. The dimethyl (2*R*,3*R*)-2-*O*-allyltartrate **1** was then reduced by LiAlH_4_ [13,14,15] or NaBH_4 _ [16,17] to give triol **2**. The two vicinal hydroxyl groups in **2** were next protected through formation of acetal **3** by the reaction with 2,2-dimethoxypropane in the presence of *p-*toluenesulfonic acid (Scheme 1). The use of the tartrates as chiral starting materials conveniently allows for the synthesis of all the possible, optically active stereoisomers of **3** and subsequently the corresponding homo-sugar analogs.

**Scheme 2:**
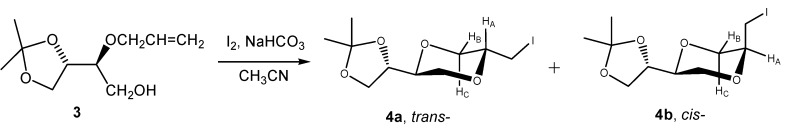
Formation of homo-sugar iodides **4a** and **4b**.

Iodocyclization of intermediate **3** in the presence of anhydrous NaHCO_3_ in dry acetonitrile [18,19,20,21] gave the 1,4-dioxane *pseudo*-sugar as a diastereomeric mixture of *trans*- and *cis*- iodides **4a** and **4b** in 26.4 % and 25.4 % isolated yields, respectively. The *trans*-compound **4a** and *cis*-compound **4b** were separated by flash chromatography using a solvent mixture of diethyl ether and *n*-hexane (gradient 1/4-1/1), Scheme 2. 

Structures **4a** and **4b** were elucidated and verified by ^1^H-, ^13^C-, and DEPT-NMR experiments in combination with 2D NMR spectroscopy techniques (COSY, HSQC, HMBC, NOESY). The assigned structures were in full agreement with the NMR data. Product **4a** was assigned the *trans*-configuration as the coupling constants *J*_AC_ and *J*_AB_ were measured to 10.2Hz and 2.4Hz, respectively. This was in agreement with the –CH_2_I group being in an equatorial position. The corresponding coupling values for the other isomer were 3.6Hz and 3.3Hz, respectively, being in agreement with the structure of the *cis*-isomer **4b**. 

**Scheme 3:**
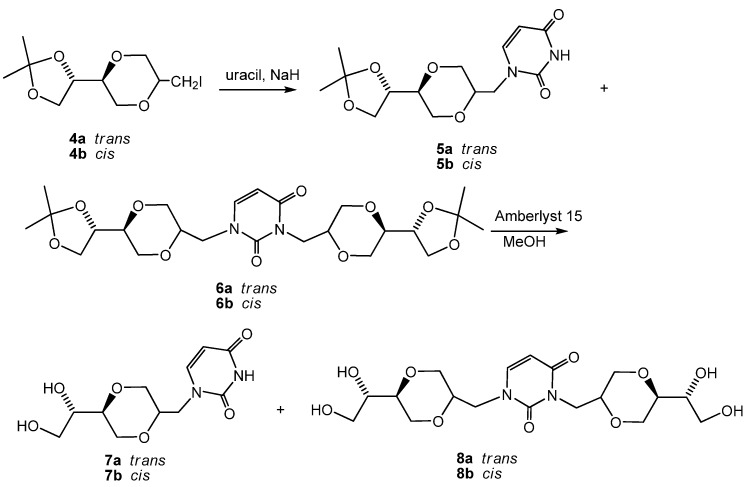
Synthesis of uracil *N*-nucleoside analogs **7a** and **7b**.

The uracil homo-*N*-nucleoside analog **5a** was obtained by reacting uracil with sodium hydride in DMF [22], followed by the reaction with *trans*-iodide **4a**. A byproduct containing two 1,4-dioxane rings was also obtained and assigned the structure **6a**. This byproduct was not easily separated from product **5a**. The acetal functions in compounds **5a** and **6a** were then removed using Amberlyst 15 in methanol providing a mixture of homo-*N*-nucleoside analog **7a** and dimer **8a**, Scheme 3. Compounds **7a** and **8a** were now readily separated by flash chromatography. The *cis*-nucleoside analogs **7b** and **8b** were obtained from **4b** by the same sequence of reactions.

**Scheme 4:**
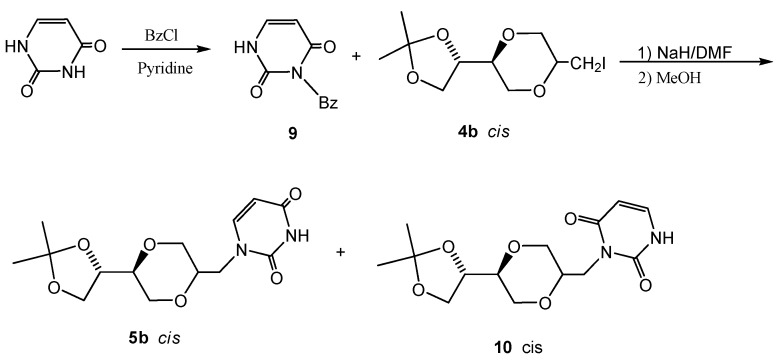
Reaction of iodide **4b** with 3-benzoyluracil, **9**.

NMR spectra of the crude reaction mixtures gave indication of an additional byproduct, which as a working hypothesis was assumed to be the corresponding 3-regioisomer **10**. To confirm the identity of the two regioisomers **5** and **10**, uracil was first selectively protected as the 3-N position by benzoylation with benzoyl chloride in pyridine to provide pure *N*-3-benzoyluracil **9**, [23]. Compound **9 **was next reacted with iodide **4b** in DMF to give product **5b** (Scheme 4). The NMR spectral data of **5b** prepared by the two different routes were in good agreement. Interestingly, the N-3 alkylation compound **10** was also observed in the product from the protected uracil **9**. A 6:5 ratio of products **5b **and **10 **was observed. The results imply that a benzoyl-walk reaction probably took place under the reaction conditions. The pure **10** was isolated from the mixture of **5b **and **10** by preparative TLC and its structure was confirmed by NMR spectroscopy. The detailed nature of these transformations was not further investigated.

**Scheme 5:**
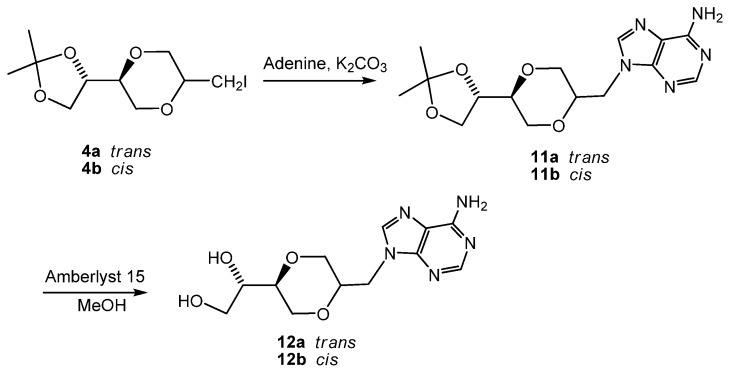
Synthesis of adenine *N*-nucleoside analogs **12a** and **12b**.

Using anhydrous potassium carbonate [24,25] as the base, the *trans*- iodide **4a** and *cis*- iodide **4b **respectively were reacted with adenine to give compound **11a** and **11b** in 42% and 30% isolated yields, respectively, after flash chromatography. Deprotection of acetals **11a** and **11b** in the presence of Amberlyst-15 in methanol gave compound **12a** and **12b **in 85% and 70% yields (Scheme 5). Different from natural occurring purine nucleosides, **12a** and **12b** were stable under acidic conditions. The depurination reaction was avoided due to the presence of the methylene group between adenine and 1,4-dioxane homo-sugar analog moiety.

**Figure 2:**
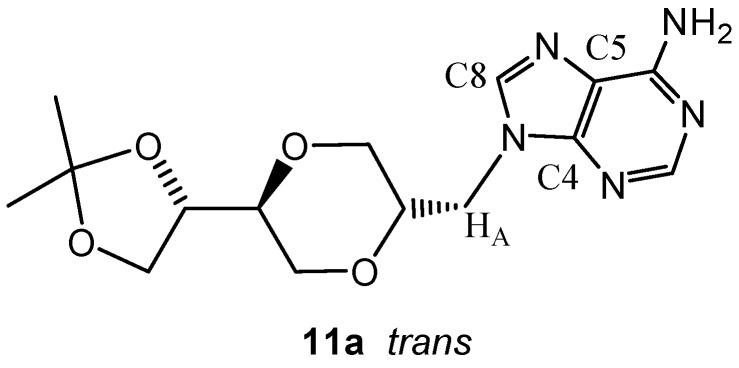
Structure of adenine homo-*N-*nucleoside analog **11a**.

The structures of the four adenine nucleoside analogs **11a**, **11b**, **12a**, **12b** were verified to be the *N*-9 adenine regioisomers by HMBC-NMR spectroscopy technique. In the case of **11a**, three bond correlations, between C4 and H_A_ and between C8 and H_A_, were observed, while three bond correlation between C5 and H_A_ was not found (Figure 2). These findings were in agreement with *e.g.* product **11a** be the N-9 adenine regioisomer.

## Conclusions

In conclusion, optically active homo-*N*-sugar nucleoside analogs containing a 1,4-dioxane moiety as the sugar analog and substituted with uracil or adenine as the base were synthesized from dimethyl tartrate. These nucleoside analogs were stable under acidic conditions. Plans for the biological screening of the produced nucleoside analogs are currently in progress. 

## Experimental

### General

NMR spectra were recorded on Bruker Avance DPX 300 or DPX 400 instruments. Chemical shifts are reported in ppm using TMS as the internal standard in CDCl_3_ or relative to 2.50 ppm for ^1^H and 39.99 ppm for ^13^C in DMSO-*d*_6_ or 3.31 ppm for ^1^H and 49.15 ppm for ^13^C in CD_3_OD. Structural assignments were based on ^1^H, ^13^C, DEPT135 and 2D spetra, COSY, HSQC, HMBC, NOESY. EI-Mass and ESI spectra were recorded on a Finnigan MAT 95XL spectrometer. IR spectra were obtained on a Thermo Nicolet FT-IR Nexus spectrometer using a Smart Endurance reflection cell. Silica gel Kieselgel 60G (Merck) was used for Flash Chromatography. The solvents were purified by standard methods. The preparations of compounds **1** were described elsewhere [9,10,11,12].

*(2S,3S)-3-(allyloxy)butane-1,2,4-triol,* (**2**)

This product was obtained by reduction of 1 with either LiAlH_4_ or NaBH_4_. *LiAlH_4_ reduction*: To a suspension of LiAlH_4_ (3.72 g, 95 %, 93 mmol) in dry diethyl ether (50 mL) was drop wise added a solution of **1** (4.36 g, 20 mmol) in 4 mL of diethyl ether at 0-5 °C. The reaction mixture was refluxed for 18 hours and then cooled in an ice bath. Then 5 mL of water was added and the mixture stirred for 20 minutes, followed by addition of a 15 % NaOH solution (12 mL) and then 10 mL of water. The resulting mixture was stirred and the granular salt formed, was separated by filtration, washed with hot THF (200 mL), and the filtrate concentrated under reduced pressure. The residue was purified by flash chromatography (CHCl_3_/CH_3_OH, 9:1 mixture) to give 0.83 g, 26 % of the pure product **2**. *NaBH_4_ reduction.* Sodium borohydride (3.45 g, 93 mmol) in ethanol (50mL) was stirred for half an hour and then dropwise added a solution of **1** (4.35 g, 20 mmol) in ethanol (15 mL). The resulting solution was refluxed gently for 5 hours. The solution was cooled in an ice bath and added 10 mL of acetic acid. The mixture was stirred for 20 minutes and filtered. The solid was washed with 2x50 ml ethanol. The combined organic phase was concentrated under reduced pressure. The crude product was purified by flash chromatography using a 19:1 mixture of CH_2_Cl_2_ / MeOH as the eluent yielding 2.88 g, 88 % of the pure product, which exhibited the following spectroscopic properties: ^1^H-NMR (CDCl_3_, 400 MHz): δ = 3.45 (q, 1H, CH-OAllyl), 3.66-3.74 (m, 3H, 1H from CH_2_-CHOH, 2H from CH_2_-CH-OAll), 3.80 (dd, 1H from CH_2_-CHOH), 3.86 (q, 1H, CHOH), 4.03-4.20 (m, 2H, OCH_2_-CH=CH_2_), 4.32 (s, broad, 3H, OH), 5.18-5.38 (m, 2H, CH_2_=CH), 5.86-5.96 (m, 1H, CH=CH_2_) ppm.^13^C-NMR (CDCl_3_, 100MHz): δ = 60.6, 63.3, 71.6, 71.8, 79.1, 117.8, 134.5 ppm. MS (EI) m/z: 145 (M^+^-OH), 131 (M^+^-CH_2_OH), 101 (OH-CH_2_=O^+^CH_2_-CH=CH_2_), 61 (HOCH_2_CH=O^+^H). IR (neat): 3365, 2881, 1736, 1448 cm^-1^.

*(S)-2-(allyloxy)-2-((S)-2,2-dimethyl-1,3-dioxolan-4-yl)ethanol* (**3**)

A solution of **2** (6.20 g, 38 mmol), 2,2-dimethoxylpropane (4.00 g, 38.5 mmol) and *p*-TsOH (223 mg, 1.2 mmol) in 100 mL acetone was stirred overnight at room temperature. The solvent was then removed and the residue was purified by flash chromatography using a 3:2 mixture of Et_2_O / *n*-hexane as the eluent to provide product **3** as acolorless oil (5.11 g, 85 %). Unreacted starting material **2** (1.05 g crude product) was recovered by continued elusion with a 19:1 mixture of CH_2_Cl_2_ / MeOH. Product **3** exhibited the following spectroscopic properties: ^1^H-NMR (CDCl_3_, 400MHz): δ = 1.37 (s, 3H, CH_3_), 1.44 (s, 1H, CH_3_), 2.48 (s, broad, 1H, OH), 3.49-3.53 (m, 1H, CH-OAll), 3.59 (dd, *J*= 10.8Hz, 12Hz, 1H, HOCH_2_-CHOAll), 3.73 (dd, *J*= 4.2Hz, 12Hz, 1H, HOCH_2_-CHOAll), 3.81 (dd, *J*= 7.2Hz, 8.4Hz, 1H, C-OCH_2_CHO-C), 4.03 (dd, *J*= 6.4Hz, 8.4Hz, 1H, C-OCH_2_CHO-C), 4.18-4.22 (m, 2H, OCH_2_-CH=CH_2_), 4.26-4.31 (m, 1H, C-OCH_2_CHO-C), 5.24-5.33 (m, 2H, CH_2_=CH), 5.88-5.95 (m, 1H, CH=CH_2_) ppm.^13^C-NMR (CDCl_3_, 100MHz): δ 25.3, 26.4, 61.6, 65.4, 71.8, 76.4, 79.1, 109.4, 117.4, 134.7 ppm. MS: (EI) m/z: 202(M^+^), 187 (M^+^-CH_3_), 171(M^+^-CH_2_OH), 101(C_5_H_9_O_2_^+^). 

*(2S,5S)-5-[(4S)-2,2-dimethyl-1,3-dioxolan-4-yl]-2-iodomethyl-1,4-dioxane* (**4a**) *and (2R,5S)-5-[(4S)-2,2-dimethyl-1,3-dioxolan-4-yl]-2-iodomethyl-1,4-dioxane* (**4b**)

To a solution of **3 **(3.20 g, 15.8 mmol) in dry acetonitrile (50 mL) was added NaHCO_3_ (4.19 g, 49.9 mmol) at -15°C. The mixture was stirred for 10 minutes and iodine (12.10 g, 47.7 mmol) was added. The reaction mixture was stirred for 68 hours with exclusion of light at -15 to -0°C. Ethyl acetate (80 mL) was added to the mixture and the solution was neutralized by saturated sodium thiosulfate solution until a colorless solution was obtained. The aqueous phase was extracted with ethyl acetate and the combined organic phase was dried over anhydrous sodium sulfate. The solution was filtered and evaporated. The residue was purified by gradient column chromatography using Et_2_O/*n*-hexane (1:4, 1:1) as eluent. The two diastereomers were separated in yields of 26.4% (**4a**) and 25.4% (**4b**). The pure compounds were white solid. R_f_ was 0.43 and 0.36 respectively (*n*-hexane/Et_2_O 1:1). The product **4a** exhibited the following spectroscopic properties: ^1^H-NMR (CDCl_3_, 400 MHz) δ = 4.10-4.02 (m, 1H, H-4”), 4.04 (dd, 1H, *J* = 11.6 Hz, 2.4 Hz, H-3_eq_), 3.97 (dd, 1H, *J*= 8.0 Hz, 6.6 Hz, H-5”), 3.79 (dd, 1H, *J*= 8.0 Hz, 6.8 Hz, H-5”), 3.79-3.53 (m, 4H, H-2, H-5’, H-6), 3.39 (dd, 1H, *J*= 11.6 Hz, 10.2 Hz, H-3_ax_), 3.07 (d, 2H, *J* = 6.0 Hz, H-7), 1.42 (d, 3 H, ^5^*J*_H-4” – H-8_ = 0.4 Hz, H-8), 1.35 (d, 3 H, ^5^*J*_H-4” – H-8_ = 0.4 Hz, H-8) ppm; ^13^C-NMR (CDCl_3_, 100 MHz) δ = 109.9, 75.4, 75.0, 74.2, 70.9, 67.9, 65.3, 26.4, 25.5, 25.4 ppm; HRMS (ESI) m/z: for C_10_H_17_IO_4_ [M+Na]^+^, Calcd. 351.0069, Found 351.0063. **4b**: ^1^H-NMR (CDCl_3_, 400 MHz): δ = 4.29 (dd, 1H, *J* = 6.8 Hz, 13.2 Hz, H-4”), 4.02 (dd, 1H, *J*= 8.0 Hz, 6.4 Hz, H-5”), 3.97 (dd, 1H, *J* = 12.0 Hz, 3.6 Hz, H-3eq), 3.84 (dd, 1H, *J*= 12.0 Hz, 3.0 Hz, H-3ax), 3.80-3.75 (m, 1 H, H-2), 3.75 (dd, 1H, *J* = 8.0 Hz, *J* = 6.8 Hz, H-5”), 3.67 (dd, 1H, *J* = 12.4 Hz, 8.0 Hz, H-6_ax_), 3.61-3.57 (m, 1H, H-5), 3.60 (dd, 1H, *J* = 12.4 Hz, 2.8 Hz, H-6_eq_), 3.42 (dd, 1H, *J* = 7.0 Hz, 12.6 Hz, H-7a), 3.40 (dd, 1H, *J* = 7.0 Hz, 13.0 Hz, H-7b), 1.44 (d, 3H, ^5^*J*_H-4” – H-8_ = 0.4 Hz, H-8), 1.38 (d, 3 H, ^5^*J*_H-4” – H-8_ = 0.4 Hz, H-8) ppm; ^13^C-NMR (CDCl_3_, 100MHz) δ = 109.8, 74.8, 73.4, 72.5, 66.3, 65.5, 62.3, 26.4, 25.3, 3.2 ppm; HRMS (ESI) m/z: for C_10_H_17_IO_4_ [M+Na]^+^, Calcd. 351.0069, Found 351.0079; IR (neat): 2980, 2867, 1461, 1413, 1380, 1370 cm^-1^.

**Figure 3:**
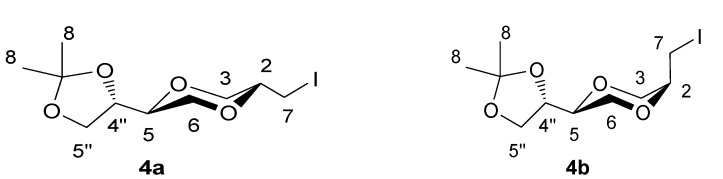
Structures **4a** and **4b**.

*(2S,5S)-5-[(4S)-2,2-dimethyl-1,3-dioxolan-4-yl]-2-(uracil-1-yl-methyl)-1,4-dioxane* (**5a**) 

To a stirred suspension of uracil (0.253 g, 2.3 mmol) in dry DMF (19 mL), sodium hydride (0.065 g, 2.7 mmol) was added at room temperature. After stirring for one hour, compound **4a** (0.35 g, 1.1 mmol) was added. The mixture was heated to 80°C and stirred overnight. The resulting mixture was evaporated under high vacuum. The residue was extracted with ethyl acetate. The solution was concentrated and purified by flash chromatography using ethyl acetate as the eluent. The product (201 mg) containing the inseparable byproduct **6a** was obtained in 60% crude yield. The product **5a** exhibited the following spectroscopic properties: ^13^C-NMR (CDCl_3_, 100 MHz): δ= 163.3, 150.9, 145.6, 109.8, 101.8, 75.2, 74.9, 73.3, 68.3, 67.4, 65.0, 48.8, 26.3, 25.2 ppm. The product contains inseparable byproduct **6a** which makes the assignments of protons difficult; IR (neat) of the mixture of **5a** and **6a**: 3214, 3093, 2983, 2869, 1659, 1453, 1054 cm^-1^; HRMS (ESI) m/z: for C_14_H_20_N_2_O_6_ [M+Na]^+^, Calcd. 335.1219, Found 335.1222. ****

**Figure 4:**
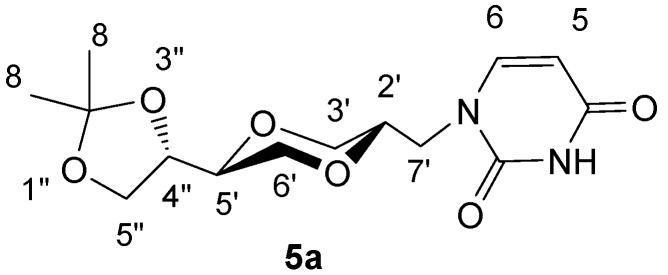
Structure **5a**.

*(2R,5S)-5-[(4S)-2,2-dimethyl-1,3-dioxolan-4-yl]-2-(uracil-1-yl-methyl)-1,4-dioxane* (**5b**)

*Method 1*: The preparation of **5b** was the same as used for the synthesis of **5a**. *Method 2*: Sodium hydride (18 mg, 0.75 mmol) in 10 mL of dry DMF was stirred for half an hour at room temperature. *N*-3-benzoyluracil **9 **(130 mg, 0.60 mmol) was added and stirred for an hour. To this suspension, iodide **4b** (98 mg, 0.30 mmol) was added. The resulting stirred mixture was heated at 90°C overnight. The mixture was concentrated under reduced pressure to remove DMF. To the residue was added 20 mL methanol and the resulting mixture was stirred for 5 minutes. The solution was concentrated and purified by flash chromatography using CH_2_Cl_2_/CH_3_OH as the eluent. The obtained product (30 mg, 32 %) containing products **5b** and **10** in a 5:6 ratio and was further purified by preparative TLC. The isolated product **5b** exhibited the following spectroscopic properties: ^1^H-NMR (CDCl_3_, 400 MHz): δ = 1.39, 1.45 (s, 2x3H, H8), 3.60 (m, 1H, H-5’), 3.67-3.74 (m, 2H, H-6’), 3.74-3.80 (m, 2H, H-5” and H-3’), 3.82-3.86 (m, 1H, H-3’), 3.91-3.96 (m, 3H, H-7’ and H-2’), 4.05 (dd, 1H, *J*= 8.2 Hz, 6.6 Hz, H-5”), 4.32-4.37 (m, 1H, H-4”), 5.70 (d, 1H, *J*= 7.8 Hz, H-5), 7.20 (d, 1H, *J*= 7.8 Hz, H-6), 8.73 (brs, 1H, NH) ppm; ^13^C-NMR (CDCl_3_, 100 MHz): δ= 163.3, 150.8, 145.1, 109.9, 102.1, 74.4, 73.8, 71.0, 65.7, 64.9, 63.3, 47.6, 26.5, 25.4 ppm; IR (neat): 3097, 2985, 2874, 1682, 1652, 1455, 1124, 1060; HRMS (ESI) m/z: for C_14_H_20_N_2_O_6_ [M+Na]^+^, Calcd. 335.1219, Found 335.1222. The product **10** exhibited the following spectroscopic properties: ^1^H-NMR (CDCl_3_, 400 MHz): δ = 1.38, 1.45 (2×3H, CH_3_), 3.41 (dd, 1H, *J* = 4 Hz, 12 Hz), 3.62-3.67 (m, 1H), 3.70-3.75 (m, 2H), 3.87 (d, 2H), 3.97-4.08 (m, 3H), 4.23-4.28 (m, 1H), 4.81 (dd, *J*= 10 Hz, 14 Hz, 1H), 5.77 (dd, *J*= 7.6 Hz, 1.6 Hz, 1H, NCH=CH), 7.16 (dd, *J*= 7.6 Hz, 5.6 Hz, 1H, NCH=CH), 8.66 (d, 1H) ppm;^ 13^C-NMR (CDCl_3_, 100MHz): δ= 25.3, 26.4, 39.8, 61.7, 65.4, 66.9, 69.1, 74.8, 76.0, 102.2, 109.6, 137.8, 151.8, 163.0 ppm; MS (m/z): (M+Na)^+^, 335.16.

**Figure 5:**
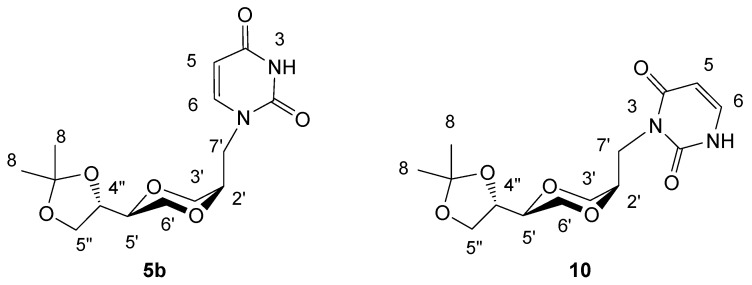
Structures **5b **and **10**.

*(2S,5S)-5-[(1S)-1, 2-dihydroxyethyl]-2-(uracil-1-yl-methyl)-1,4-dioxane* (**7a**)

The mixture of **5a **and **6a** (60mg, 5/6 ratio) was dissolved in methanol (10 mL), Amberlyst 15 (50 mg) was added and the mixture refluxed. The reaction was monitored by TLC until no more **7a** was observed. The solution was filtered and the solvent was evaporated. The obtained product was further purified by flash chromatography to give pure **5a** (12 mg, 34 %) and **8a **(25 mg, 38 %) using CH_2_Cl_2_/MeOH (13:1) as eluent. The product **7a** exhibited the following spectroscopic properties: ^1^H-NMR (CD_3_OD, 400 MHz): δ = 3.32-3.38 (m, 1H, H-3’), 3.47-3.55 (m, 2H, H-1” and H-2”), 3.55-3.67 (m, 4H, H-5’, H-2”, H-6’ and H-7’), 3.74-3.80 (m, 1H, H-2’), 3.82 (dd, 1H, *J*= 1.2 Hz, 10.4 Hz, H-6’), 3.88 (dd, 1H, *J*= 2.8 Hz, 8.4 Hz, H-3’), 3.91 (dd, 1H, *J*= 3 Hz, 11 Hz, H-7’), 5.61 (d, 1H, *J*= 8 Hz, H-5), 7.52 (d, 1H, *J*= 8 Hz, H-6) ppm; ^13^C-NMR (CD_3_OD, 100MHz): δ = 50.0, 63.7, 69.3, 69.7, 72.6, 74.4, 76.6, 101.8, 148.5, 153.0, 166.9 ppm; IR (neat): 3396, 2871, 1651, 1455, 1101, 1043 cm^-1^; HRMS (ESI) m/z: for C_11_H_16_N_2_O_6_ [M+Na]^+^, Calcd. 295.0906, Found 295.0911.

**Figure 6:**
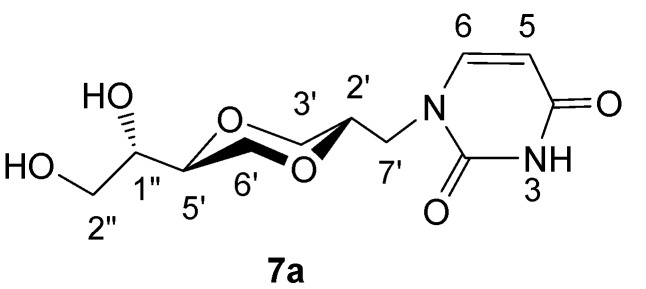
Structure **7a**.

The product **8a** exhibited the following spectroscopic properties: ^1^H-NMR (CD_3_OD, 400 MHz): δ = 3.34-3.43 (m, 2H), 3.46-3.54 (m, 4H), 3.57-3.74 (m, 8H), 3.75-3.86 (m, 6H), 3.87-3.90 (m, 1H), 3.92-3.96 (m, 1H), 4.05-4.10 (m, 1H), 5.70 (d, 1H, *J*= 7.8 Hz, NCH=CH), 7.52 (d, 1H, *J*= 7.8 Hz, NCH=CH) ppm; ^13^C-NMR (CD_3_OD, 100 MHz): δ = 42.8, 51.1, 63.7, 63.8, 69.3, 69.4, 69.7, 72.57, 72.61, 74.0, 74.4, 76.56, 76.57, 101.2, 146.8, 153.3, 165.6 ppm; MS (m/z): HRMS (ESI) m/z: for C_18_H_28_N_2_O_10_ [M+Na]^+^, Calcd. 455.1641, Found 455.1645.

*(2R,5S)-5-[(1S)-1,2-dihydroxyethyl]-2-(-uracil-1-yl-methyl)-1,4-dioxane* (**7b**) 

The method for preparation of **7b** was as same as applied for the synthesis of **7a**. The product **7b** exhibited the following spectroscopic properties: ^1^H-NMR (CD_3_OD, 400 MHz): δ= 3.53-3.66 (m, 3H, H-2” and H-6’), 3.60-3.70 (m, 2H, H-1” and H-5’), 3.78-3.80 (m, 2H, H-3’), 3.80-3.92 (2H, H-2’ and H-7’), 4.00 (dd, 1H, *J*= 11.6 Hz, 8 Hz, H-2” or H-6’), 4.23-4.31 (m, 1H, H-7’), 5.65 (d, *J*= 8 Hz, H-5), 7.58 (d, *J*= 8 Hz, H-8) ppm; ^13^C-NMR (CD_3_OD, 100 MHz): δ= 166.8, 153.0, 147.9, 102.2, 76.4, 71.8, 71.5, 66.9, 63.9, 63.8, 48.2 ppm; IR (neat): 3352, 3056, 2931, 2875, 1667, 1456, 1129, 1101 cm^-1^; HRMS (ESI) m/z: for C_11_H_16_N_2_O_6_ [M+Na]^+^, Calcd. 295.0906, Found 295.0915.

**Figure 7:**
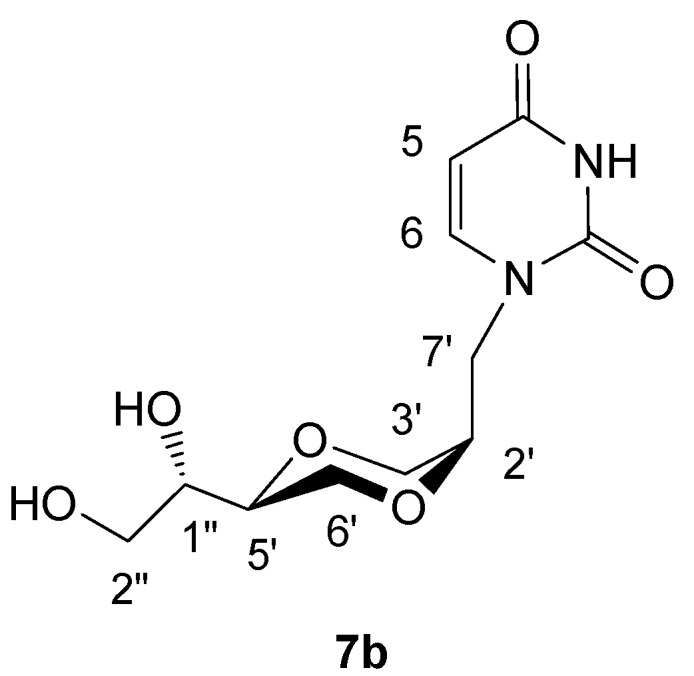
Structure **7b**.

*(2S,5S)-5-[(4S)-2,2-dimethyl-1,3-dioxolan-4-yl]-2-(adenin-9-yl-methyl)-1,4-dioxane* (**11a**)

A mixture of adenine (0.279 g, 2 mmol) and anhydrous potassium carbonate (0.300 g, 2.2 mmol) in dry DMF (10 mL) was heated at 120°C. After two hours, compound **4a** (0.304 g, 0.9 mmol) was added to the solution. The mixture was stirred overnight at 120°C. The mixture was concentrated under reduced pressure and the residue purified by flash chromatography using a mixture of dichloromethane and methanol (14:1) as the eluent. The product (130 mg) was obtained in 42 % yield. The product **11a** exhibited the following spectroscopic properties: ^1^H-NMR (CDCl_3_, 400 MHz): δ= 8.35 (s, 1H, H-2), 7.88 (s, 1H, H-8), 5.75 (s, 2H, NH_2_), 4.29 (dd, 1H, *J*= 14.6 Hz, 3.4 Hz, H-7’), 4.12 (dd, 1H, *J*= 14.6 Hz, 6.8 Hz, H-7’), 4.01- 4.07 (m, 1H, H-4”), 3.97 (dd, *J*= 11.4 Hz, 2.6 Hz, H-3’eq), 3.87-3.95 (m, 2H, H-2’, H-5”), 3.75-3.82 (m, 2H, H-5”, H-6’), 3.46-3.57 (m, 2H, H-5’, H-6’), 3.32 (dd, *J*= 11.4 Hz, 10.6 Hz, H-3’ax), 1.39 (s, 1H, H-10), 1.33 (s, 1H, H-10) ppm. ^13^C-NMR (CDCl_3_, 100 MHz): δ= 155.6, 153.2, 150.4, 141.6, 141.1, 119.4, 109.7, 75.1, 74.9, 73.2, 68.5, 67.5, 65.0, 44.2, 26.2, 25.3 ppm; IR (neat): 3322, 3161, 2983, 2938, 2864, 1673, 1606, 1064, 1048 cm^-1^; HRMS (ESI) m/z: for C_15_H_22_N_5_O_4_ M^+^, Calcd. 336.1671, Found 336.1676.

**Figure 8:**
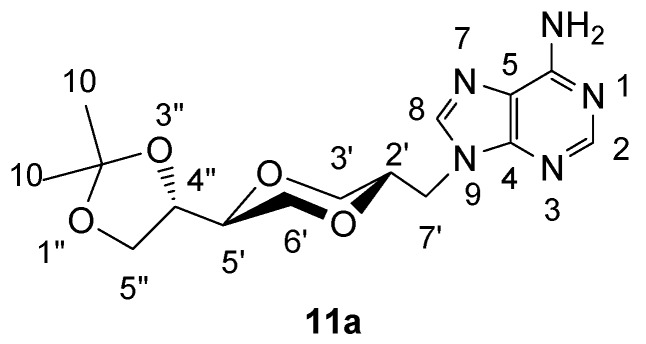
Structure **11a**.

*(2R,5S)-5-[(4S)-2,2-dimethyl-1,3-dioxolan-4-yl]-2-(adenin-9-yl-methyl)-1,4-dioxane* (**11b**)

Synthesis of **11b** was carried out as described for **11a**. The product exhibited the following spectroscopic properties: ^1^H-NMR (CDCl_3_, 400 MHz): δ= 8.35 (s, 1H, H-2), 7.87 (s, 1H, H-8), 6.14 (s, 2H, NH_2_), 4.54 (dd, 1H, *J*= 14.8 Hz, 8.8 Hz, H-7’), 4.32-4.38 (m, 2H, H-4”, H-7’), 4.06- 4.11 (m, 1H, H-2’), 4.05 (dd, *J*= 8.4 Hz, 6.6 Hz, H-5”), 3.80-3.90 (m, 3H, H-3’, H-6’), 3.77 (dd, *J*= 8.4 Hz, 6.8 Hz, H-5”), 3.61-3.67 (m, 2H, H-5’, H-6’), 1.46 (s, 1H, H-10), 1.39 (s, 1H, H-10) ppm; ^13^C-NMR (CDCl_3_, 100 MHz): δ= 155.7, 153.1, 150.1, 140.9, 119.4, 109.9, 74.6, 73.8, 71.1, 65.6, 65.1, 63.0, 42.8, 26.5, 25.4 ppm; IR (neat): 3276, 3134, 2984, 2935, 2870, 1676, 1600, 1575, 1126, 1066 cm^-1^; HRMS (ESI) m/z: for C_15_H_22_N_5_O_4_ M^+^, Calcd. 336.1671, Found 336.1675.

**Figure 9:**
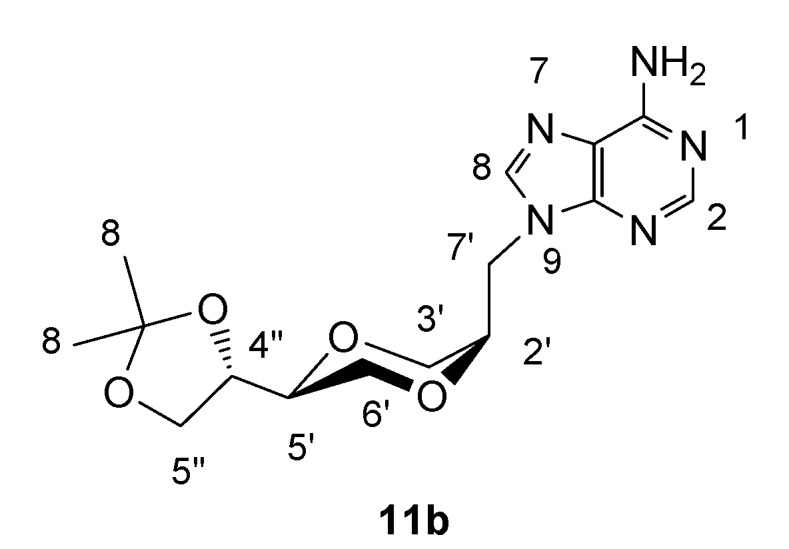
Structure **11b**.

*(2S,5S)-5-[(1S)-1,2-dihydroxyethyl]-2-(adenin-9-yl-methyl)-1,4-dioxane* (**12a**)

Compound **11a** (108 mg, 0.32 mmol) was dissolved in methanol (10 mL), Amberlyst 15 (45 mg) was added and the mixture was refluxed until TLC showed that all **11a** was consumed. The solution was filtered and the solvent was evaporated. The product **12a** (81 mg, 85 %) exhibited the following spectroscopic properties: ^1^H-NMR (DMSO-*d*_6_, 400 MHz): δ= 8.17 (s, 1H, H-2), 8.08 (s, 1H, H-8), 7.35 (brs, 2H, NH_2_), 4.3-4.8 (brs, 2H, OH), 4.21 (dd, 1H, *J*= 14.4 Hz, 4.2 Hz, H-7’), 4.13 (dd, 1H, *J*= 14.4 Hz, 6.8 Hz, H-7’), 3.83-3.88 (m, 2H, H-2’, H-3’), 3.67-3.73 (m, 1H, H-6’), 3.17-3.48 (m, 6H, H-6’, H-5’, H-3’, H-1”, H-2”) ppm; ^13^C-NMR (DMSO-*d*_6_, 100 MHz): δ= 155.5, 151.9, 149.6, 141.6, 118.4, 75.2, 72.6, 70.8, 68.1, 67.5, 61.9, 43.7 ppm; IR (neat): 3271, 3117, 2918, 2881, 1668, 1604, 1120, 1106, 1066 cm^-1^; HRMS (ESI) m/z: for C_12_H_17_N_5_O_4_ [M+1]^+^, Calcd. 296.1358, Found 296.1356.

**Figure 11:**
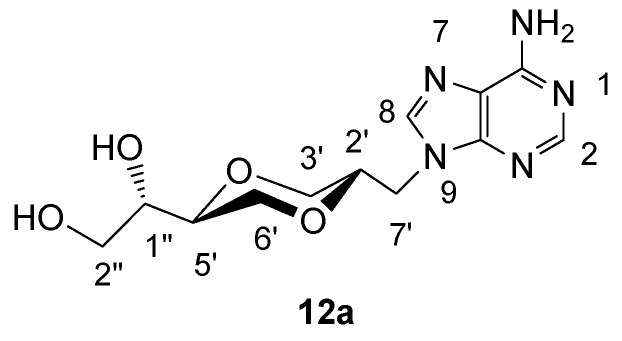
Structure **12a**.

*(2R,5S)-5-[(1S)-1,2-dihydroxyethyl]-2-(adenin-9-yl-methyl)-1,4-dioxane* (**12b**)

Compound **12b** was prepared using the same method as described for the synthesis of **12a**. Thus, **11b** (79 mg, 0.24 mmol) in methanol (10 mL) containing added Amberlyst-15 (36 mg) was refluxed until TLC showed that all **11b** was consumed. The solution was filtered and the solvent was evaporated. The product **12b **(49 mg, 70%) exhibited the following spectroscopic properties:^ 1^H-NMR (DMSO-*d*_6_, 400 MHz): δ= 8.16 (s, 1H, H-2 or H-8), 8.15 (s, 1H, H-8 or H-2), 7.30 (brs, 2H, NH_2_), 4.69 (dd, 1H, *J*= 14.4 Hz, 9.6 Hz, H-7’), 4.22 (dd, 1H, *J*= 14.4 Hz, 4.2 Hz, H-7’), 4.00-4.05 (m, 1H, H-2’), 3.92-3.98 (m, 1H, H-2” or H-6’), 3.78 (dd, 1H, *J* = 12.2 Hz, 2.2 Hz, H-3’), 3.69 (dd, 1H, *J*= 12.2 Hz, 3.2 Hz, H-3’), 3.34-3.56 (m, 5H, remining protons) ppm; ^13^C-NMR (DMSO-d_6_, 100MHz): δ= 155.7, 152.0, 149.6, 141.3, 118.5, 75.5, 70.4, 69.6, 65.6, 62.2, 61.4, 40.6 ppm; IR (neat): 3271, 3125, 2883, 1674, 1604, 1119, 1065 cm^-1^; HRMS (ESI) m/z: for C_12_H_17_N_5_O_4_ [M+1]^+^, Calcd. 296.1358, Found 296.1355.

**Figure 10:**
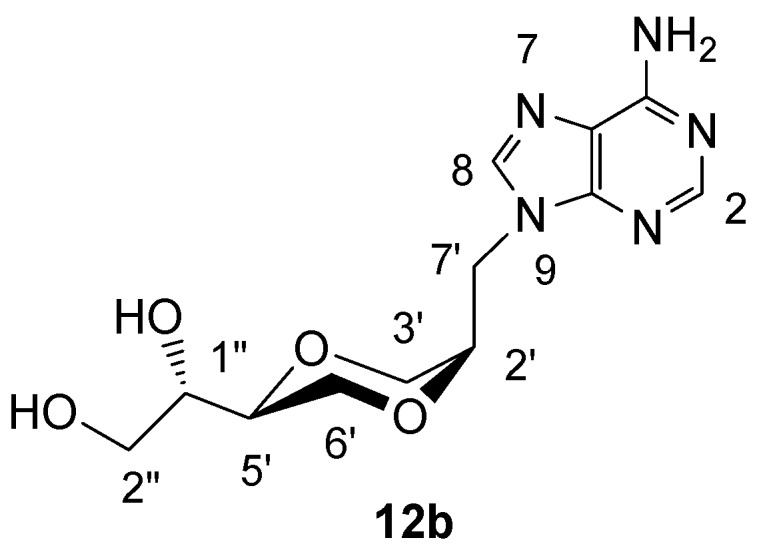
Structure **12b**.
